# Analgesic Tolerance Development during Repetitive Electric Stimulations Is Associated with Changes in the Expression of Activated Microglia in Rats with Osteoarthritis

**DOI:** 10.3390/biomedicines8120575

**Published:** 2020-12-07

**Authors:** Suk-Chan Hahm, Jin Seung Lee, Young Wook Yoon, Junesun Kim

**Affiliations:** 1 Graduate School of Integrative Medicine, CHA University, Seongnam 13488, Korea; schahm@cha.ac.kr; 2 Department of Physical Therapy, Korea University College of Health Science, Seoul 02841, Korea; jslee0320@korea.ac.kr; 3 Rehabilitation Science Program, Department of Health Sciences, Graduate School, Korea University, Seoul 02841, Korea; 4 BK21FOUR R&E Center for Learning Health Systems, Korea University, Seoul 02841, Korea; 5 Department of Physiology, Korea University College of Medicine, Seoul 02841, Korea; ywyoon@korea.ac.kr

**Keywords:** microglia, osteoarthritis, pain, electric stimulation, transcutaneous electrical nerve stimulation, tolerance

## Abstract

Electric stimulation is used for managing osteoarthritic (OA) pain; however, little is known about the development of analgesic tolerance during repeated stimulations and the relation of spinal microglia with OA pain. We investigated the changes in the analgesic effects of repeated electric stimulations and the relation between the development of analgesic tolerance and spinal microglial expression in rats with OA. To induce OA, monosodium iodoacetate was injected into the synovial space of the right knee joint of the rats (*n* = 185). Repeated high frequency, low frequency, or sham transcutaneous electric nerve stimulation (TENS) was performed to the ipsilateral knee joint for 20 min in rats with OA (*n* = 45). Minocycline or minocycline plus TENS (HF, LF, or sham) was treated in OA rats with repeated TENS-induced tolerance (*n* = 135). Immunohistochemistry of the microglia in the L3–L5 spinal segments was performed. Knee joint pain during passive movement of the knee joint were quantified using the knee-bend score and the proportion of activated microglia was calculated as primary variables. Paw withdrawal threshold (hypersensitivity to mechanical stimuli) was assessed and the resting and activated microglia were counted as secondary variables. Repeated applications decreased the analgesic effect of TENS on OA pain and failed to reduce the expression of activated microglia in the spinal cord. However, spinal microglial inhibition by minocycline restored the analgesic effect of TENS on OA pain in TENS-tolerant OA rats. TENS combined with minocycline treatment improved knee joint pain and mechanical hypersensitivity in TENS-tolerant OA rats, and inhibited the expression of activated microglia in the spinal cord. These results suggest a possible relationship between repetitive electric stimulation-induced analgesic tolerance for OA pain control and changes in microglia activation.

## 1. Introduction

Worldwide, approximately 40% of adults aged 65 and older suffer from chronic pain and dysfunction due to osteoarthritis (OA) [1]. Electrotherapy, in particular, transcutaneous electrical nerve stimulation (TENS) is a method to manage OA and several clinical studies have reported the effects of TENS on OA pain [2,3,4,5]. Electrical stimulus by TENS is delivered to the skin area where the electrode is attached, and a tingling sensation is felt. Several previous studies have reported mechanisms for TENS induced analgesia including decreased spinal glial expression [6], increased opioid release and opioid receptor activation in the spinal or supra-spinal area [7,8,9], decreased aspartate and glutamate release in the spinal cord [10], increased spinal serotonin release and serotonin receptor activation [11,12], and increased γ-aminobutyric acid (GABA) release and GABA_A_ receptor activation in the spinal cord [13]. However, some long-term TENS users experienced a decline in the efficacy of TENS in several chronic pain conditions [14]. Furthermore, a study has reported that repeated TENS applications decrease the analgesic effect [15].

Repeated administration of morphine or opioid agonists decreases their analgesic effect called opioid tolerance [16]. Studies have reported changes in glial responses to opioids such as morphine-induced upregulation of spinal glial cells [17,18,19]. Pro-inflammatory mediators released by spinal glial cells in several pathological conditions increase the excitability of spinal neurons, which affect chronic pain [20,21,22]. Glial activation induced by chronic opioid treatment reduces opioid analgesia and contributes to the development of tolerance [23]. Moreover, Ueda et al. has reported that increased glial activity due to anti-opioid system activation contributes to opioid tolerance [24].

Several studies have reported that the TENS induced analgesia is generated by release of endogenous opioids or the activation of opioid receptors in the central nervous system [7,8,9]. Patients who had used opioids long enough to establish tolerance did not significantly respond to TENS [25]. Considering opioid release and opioid receptor activation as a mechanism of analgesic TENS effect [7,8,9] and clinical evidence for no analgesic effect of TENS by opioid tolerance [20], if the analgesic effects of TENS on OA pain is reduced by repeated TENS applications, decrease of analgesic TENS effects may share the mechanisms in opioid tolerance.

According to a recent study [6], TENS effectively reduced OA pain, and its effect was related to the decreased expression of activated microglia in the spinal cord. If repeated TENS applications fail to control OA pain, namely, the development of TENS-induced analgesic tolerance, it may be related to changes in spinal glial activity. To date, no in vivo study has been specifically conducted to investigate the changes in the effects of repeated TENS applications on OA pain and the relation between TENS-induced analgesic tolerance and microglial expression. Thus, this study examines the changes in the analgesic effects of repeated high frequency (HF), low frequency (LF), or sham TENS applications and changes in the expression of spinal microglia in rats with OA. In addition, we investigated the effect of minocycline (microglia inhibitor) [26,27] or TENS (HF, LF, or sham) along with minocycline treatment on OA pain and the changes in the expression of spinal microglia in OA rats with repeated TENS-induced analgesic tolerance.

## 2. Materials and Methods

### 2.1. Experimental Animals

Adult male Sprague–Dawley rats (*n* = 185, Orient Bio Inc., Seoul, Korea) with weights ranging from 200 g to 220 g were used in this study. The animals were kept in a 12-h light and 12-h dark cycle. They were kept for seven days before drug injection for OA induction with access to water and food *ad libitum*. This study was approved by the Institutional Animal Care and Use Committee (Approval number, date: KUIACUC-2016-95, 2 April 2016) of Korea University, and all experimental procedures were performed in accordance with the institution’s guidelines.

### 2.2. Induction of OA

In this study, monosodium iodoacetate (MIA)-induced OA animal model which mimics ongoing human OA pain [28,29], characterizes OA pain associated with OA structural severity and synovitis [30], and shows increase of glial cell expression [27] related to OA pain after injection was used.

To induce OA, 4 mg of monosodium iodoacetate (MIA) in 50-μL saline (Millipore Sigma, St. Louis, MO, USA) was intra-articularly injected into the right knee joint cavities (synovial space) of rats under light anesthesia (2% isoflurane in O_2_) [6,31]. After injection, repeated flexion–extension of the injected knee joint was performed to maximize MIA diffusion. The animals were allowed to recover from the effects of anesthesia for 10 min before returning them to their cages.

### 2.3. TENS Application

Under 2% isoflurane (Hana Pharm Co., Seoul, Korea) anesthesia, the hair on the right knee joint of the rats was shaved, and electrodes (1 cm in diameter) were placed on the medial and lateral aspects of the joint. TENS (Chattanooga Group Inc., Chattanooga, TN, USA) was applied. Sham, HF (100 Hz), or LF (4 Hz) TENS was applied as follows: intensity, sensory level (just below the motor contraction level); pulse width, 100 μs; application type, constant mode; wave form, an asymmetrical biphasic waveform; and application time, 20 min [3,6,8,13]. The sham TENS group had skin electrodes attached but received no electric delivery.

### 2.4. Intrathecal Catheterization

For intrathecal drug delivery, a sterilized polyethylene-10 tube (Becton Dickinson and Company, Franklin Lake, NJ, USA) was inserted into the subarachnoid space (catheter length: 8.5 cm) toward the lumbar enlargement under 3% isoflurane anesthesia. All animals were allowed to recover for one week before the experiments. Rats with limb paralysis or paresis after intrathecal catheterization were excluded and euthanized.

### 2.5. Drug Delivery

To investigate the effects of spinal microglia inhibition and the effects of the combination of microglial inhibition and TENS treatment on OA pain in TENS-tolerant rats, 100, 30, and 10 μg/10 μL of minocycline (Millipore Sigma, St. Louis, MO, USA) in artificial cerebrospinal fluid were injected intrathecally, respectively.

### 2.6. Behavioral Analyses

Knee joint pain during passive movement of the knee joint were quantified using the knee-bend score as a primary variable for OA pain. Paw withdrawal threshold (hypersensitivity to mechanical stimuli) was assessed as a secondary variable for OA pain.

The paw withdrawal threshold (PWT) is the threshold of brisk paw withdrawal response to a graded mechanical stimulus and was assessed using a series of eight von Frey filaments (Stoelting Co., Wood Dale, IL, USA). Fifty percent of the withdrawal threshold was determined using the up–down method [32] while initiating a 2.0-g (4.31 mN) strength filament in the middle of the series of eight von Frey filaments with a logarithmically increasing stiffness (0.41–15.10 g). The 50% of the withdrawal threshold was interpolated according to the method described by Dixon [33].

To quantify pain for normal knee joint movement in rats with OA, the knee-bend test was conducted [34]. Briefly, each test consisted of five flexions and five extensions of the knee joint, and the total number of vocalizations or struggle was recorded. A score of 0 means no responses to any kind of extension or flexion of the joint, and a score of 0.5 was given for struggling with maximal flexion/extension. A score of 1 was given for struggling with moderate flexion/extension and for vocalizations to maximal flexion/extension. A score of 2 indicated squeaks in response to moderate flexion/extension of the joint. The knee-bend score (KBS) was the sum of the aforementioned scores with a maximum value of 20.

The area under the curve (AUC) of PWT or KBS was calculated for comparison of the overall analgesic effect of TENS among the groups using the following formula [6]:

$$\overset{6}{\underset{i=1}{\sum }}\left|\;\left\{\left(V_{0}-V_{i}\right)+\left(V_{0}-V_{i-1}\right)\right\}\;\right|*\Delta t÷2$$

where *V*_0_ indicates pre-value; *V_i_* indicates post-value at each time point (*i* = 1, 2, 3, 4, 5, and 6→20, 40, 60, 80, 100, and 120 min after TENS, respectively); and Δ*t* is the time frame between the assessments (20 min).

### 2.7. Tissue Processing and Immunohistochemistry

Tissues were extracted from the lumbar (L3–L5) segments. The rats were deeply anesthetized using urethane (1.25 g/kg, intraperitoneal injection) and perfused intracardially with 0.9% saline and 0.01% heparin, followed by 4% buffered paraformaldehyde. The tissues were postfixed for one day in 4% paraformaldehyde and preserved for three days at 4°C in 30% sucrose. Cryosections (14 μm) from each group were obtained.

Spinal cord tissues were first blocked using avidin and biotin for 15 min each and then incubated overnight with a primary antibody: mouse anti-CD11b for microglia (1:2000; Bio-Rad Laboratories, Inc., Oxford, UK). Subsequently, the sections were incubated with biotinylated goat anti-mouse IgG (1:1000, Vector Laboratories, Inc., Burlingame, CA, USA), a secondary antibody, and strep-488 for microglia (1:1000; Invitrogen, Thermo Fisher Scientific Inc., Carlsbad, CA, USA) for one hour each.

### 2.8. Quantitative Image Analysis

Quantitative analysis was performed by an experimenter blinded to the treatment groups using Leica analysis software (Leica Microsystems GmbH, Wetzlar, Germany). The stained sections were examined using a Leica DM 2500 fluorescence microscope (Leica Microsystems GmbH, Wetzlar, Germany), and images were captured using a Leica camera DFC 450C (Leica Microsystems GmbH, Wetzlar, Germany).

The proportion of activated microglia was calculated as a primary variable for spinal microglia expression. The resting and activated microglia were counted as secondary variables for spinal microglia expression. The resting and activated microglia were classified based on the method described in previous studies [6,22,35]. The resting and activated microglia were counted in the superficial dorsal horn (laminae I–III) of the L3–L5 segments, and the proportion of activated microglia (number of activated microglia/total number of microglia) was calculated.

### 2.9. Experimental Procedure

**Figure 1:**
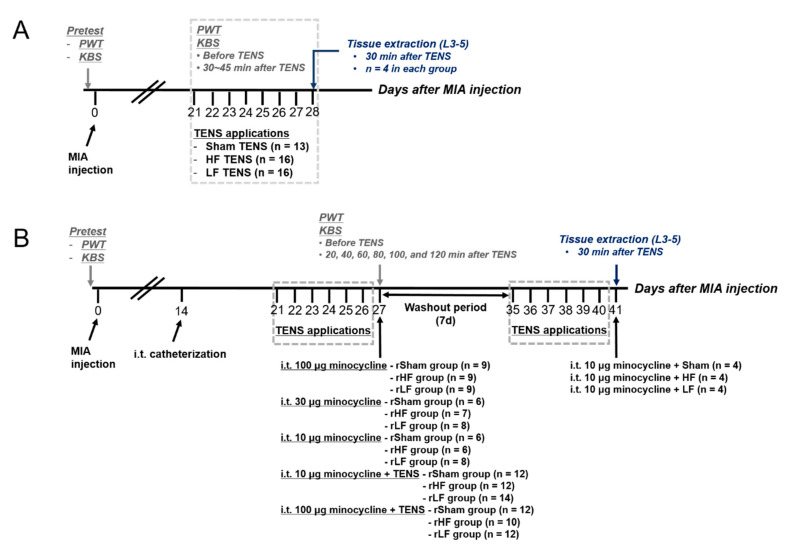
Diagram of the timelines of the two experiments (**A**) Experimental design for the effect of repeated transcutaneous electric nerve stimulation (TENS) application on osteoarthritic pain and changes in the expression of spinal microglia in rats with oseteoarthritis. (**B**) Experimental design for the effect of microglia inhibition or TENS with microglia inhibition on osteoarthritic pain changes in the expression of spinal microglia in TENS-tolerant rats. PWT, paw withdrawal threshold; KBS, knee bend test; MIA, monosodium iodoacetate; HF, high frequency; LF, low frequency; rHF, rats developed tolerance to the analgesic effect of TENS due to repeated HF TENS applications; rLF, rats developed tolerance to the analgesic effect of TENS due to repeated LF TENS applications; rSham, rats with repeated sham TENS applications (no tolerance development).

In this study, all experimental procedures consisted of two steps. First, changes of analgesic TENS effects on OA pain and changes in spinal glial cells due to repeated TENS applications were investigated (Figure 1A). MIA was injected into the right knee joint cavity of the rats (*n* = 45). Before repeated TENS applications, the rats were randomly allocated into three groups using a simple randomization method 21 days after MIA injection. In all behavior tests, the assessor was blinded to the experimental groups. Another experimenter applied sham (*n* = 13), HF (*n* = 16), or LF TENS (*n* = 16) on the ipsilateral knee joint of the rats with OA once a day for eight days 21 days after MIA injection. PWT and KBS were measured daily before TENS application and 30–45 min after TENS application. L3–L5 segments were extracted from four randomly selected rats 30 min after eight days of sham, HF, or LF TENS applications from each group (28 days after MIA injection). Rats excluded from tissue extraction were euthanized. Second, the effects of microglia inhibitor (minocycline) or TENS combined with microglia inhibitor on OA pain and the changes in the expression of spinal microglia in OA rats with TENS-induced analgesic tolerance were investigated (Figure 1B). MIA was injected into the right knee joint cavity of the rats (*n* = 140). Intrathecal catheterization was performed for the rats with induced OA 14 days after MIA injection. Before TENS applications to develop TENS-induced tolerance, the rats were randomly allocated into three groups using simple randomization method 21 days after MIA injection, and TENS was applied once a day for six days (analgesic tolerance developed after six applications in the first experiment). In all behavior tests, the assessor was blinded to the experimental groups. Another experimenter intrathecally administered minocycline to rats with OA with TENS tolerance. To investigate the effect of TENS with microglia inhibition on OA pain in TENS-tolerant rats, minocycline was administered and then sham, HF, and LF TENS were applied. PWT and KBS were measured before delivery and TENS application and 20, 40, 60, 80, 100, and 120 min after drug delivery and TENS application. All rats had a washout period of seven days (our team assessed, and rats with KBS and PWT values close to the mean were randomly allocated into the groups, and the L3–L5 segments were extracted 30 min after TENS with minocycline treatment (*n* = 4, in each group). Rats excluded from tissue extraction were euthanized.

### 2.10. Statistics

All values were expressed as the mean ± standard error of the mean. The histogram was checked to determine whether each variable was normally distributed. In this study, nonparametric tests were used because distribution of variables assessed in this study were not symmetric or it was difficult to confirm the distribution of the variable due to small sample. The Friedman test followed by a post-hoc analysis (Wilcoxon signed-rank test with the Bonferroni correction) was used to analyze the behavioral changes over time. The Kruskal–Wallis test followed by a post-hoc analysis (Mann–Whitney U test with the Bonferroni correction) was performed to investigate the behavioral differences and changes in glial cells among groups. *p*-values of < 0.05 were used to denote statistical significance. All statistical analyses were performed using Statistical Package for the Social Sciences (version 21.0; IBM Corp., Armonk, NY, USA).

## 3. Results

### 3.1. Changes of OA Pain by Repeated TENS in Rats with OA

**Figure 2:**
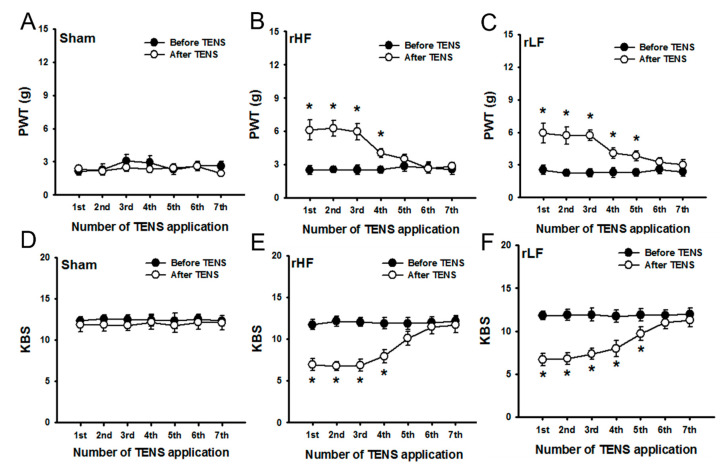
The effects of repeated transcutaneous electric nerve stimulation (TENS) applications on mechanical hypersensitivity in the hind paw and knee joint pain to passive movement in rats with osteoarthritis (**A**) The paw withdrawal threshold (PWT) of the ipsilateral side did not change following repeated TENS applications in the sham TENS group. The PWT in the high-frequency (HF) (**B**) and low-frequency (LF) TENS (**C**) groups significantly changed following the 1st–4th applications and 1st–5th applications of TENS, respectively, compared with that before TENS application. (**D**) The knee-bend score (KBS) of the ipsilateral side did not change following repeated TENS applications in the sham TENS group. However, the KBS in the HF (**E**) and LF TENS (**F**) groups significantly changed after the 1st–4th and 1st–5th applications of TENS, respectively, compared with that before TENS application. Asterisks (*) indicate values significantly different from the pre-TENS value.

To investigate whether repetitive TENS application could induce analgesic tolerance on OA pain, TENS was applied daily. PWT and KBS values were assessed before and after TENS application. No significant improvements in PWT and KBS were observed before and after repeated sham TENS application (Figure 2A,D), and repeated TENS-induced tolerance on OA pain was not observed in the sham TENS group. HF TENS showed a significant reduction of MIA-induced increased mechanical sensitivity to mechanical stimuli in the hind paw and a significant reduction in knee joint pain following the first and fourth TENS applications compared to those before TENS application; however, repeated applications of HF TENS decreased the analgesic effect on OA pain after five applications (Figure 2B,E). LF TENS significantly reduced mechanical hyperalgesia to mechanical stimuli in the hind paw and knee joint pain after the fifth TENS application compared with those before the application of TENS (Figure 2C,F). However, no significant reduction in PWT and knee joint pain was observed after the sixth LF TENS application compared with those before TENS application (Figure 2C,F). A tolerance-like effect of TENS (repeated TENS-induced tolerance) on OA pain was observed in both the HF TENS and LF TENS groups.

### 3.2. Changes in Spinal Microglia due to Repeated TENS Applications in Rats with OA

**Figure 3:**
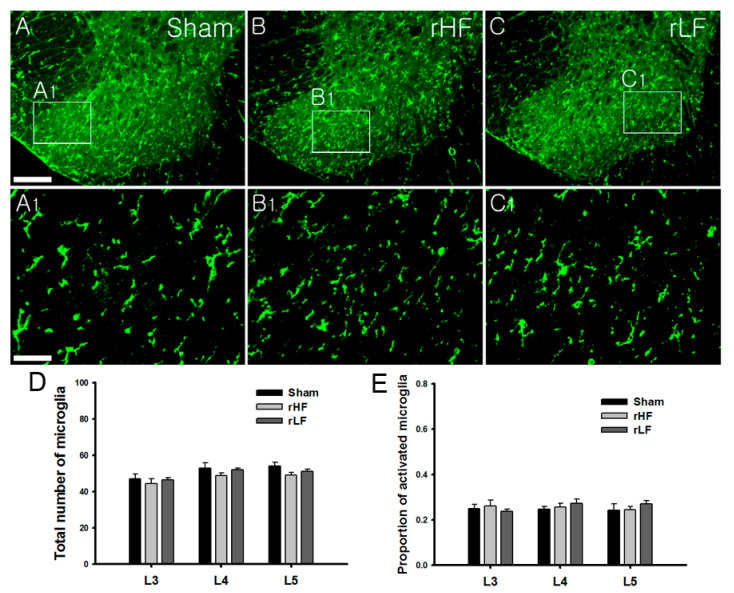
Changes in the expression of spinal microglia after repeated transcutaneous electric nerve stimulation (TENS) applications (**A**,**A_1_**) The expression of activated microglia was exhibited after repeated sham TENS application. Moreover, repeated high-frequency (HF) TENS (**B**,**B_1_**) and repeated low-frequency (LF) TENS (**C**,**C_1_**) showed the expression of the activated microglia in the spinal cord. (**D**,**E**) HF and LF TENS did not significantly change the proportion of activated microglia and the total number of microglia expressed in the L3–L5 segments compared with sham TENS. Scale bar: (**A**–**C**) 200 μm and (**A_1_**,**B_1_**,**C_1_**) 50 μm.

To determine whether repeated TENS application was associated with changes in spinal microglia, the expression of spinal microglia was investigated. Eight days after TENS application, the animals were euthanized immediately for immunohistochemistry. A recent study has shown a significant reduction in the proportion of activated microglia after HF or LF TENS application [6]. In this study, the repeated sham TENS group showed an expression of an activated microglia phenotype with a marked cellular hypertrophic appearance (Figure 3A,A_1_). Moreover, both the repeated HF (Figure 3B,B_1_) and LF TENS (Figure 3C,C_1_) groups showed an expression of activated microglia phenotype, which is similar to that in the sham TENS group. In the analysis of the number of spinal microglia, repeated HF and LF TENS did not significantly change the total number of microglia compared with sham TENS (Figure 3D). Compared with sham TENS, repeated HF and LF TENS did not significantly reduce the proportion of activated microglia (Figure 3E).

### 3.3. Effects of Microglial Inhibition on OA Pain in TENS-Tolerant Rats

**Figure 4:**
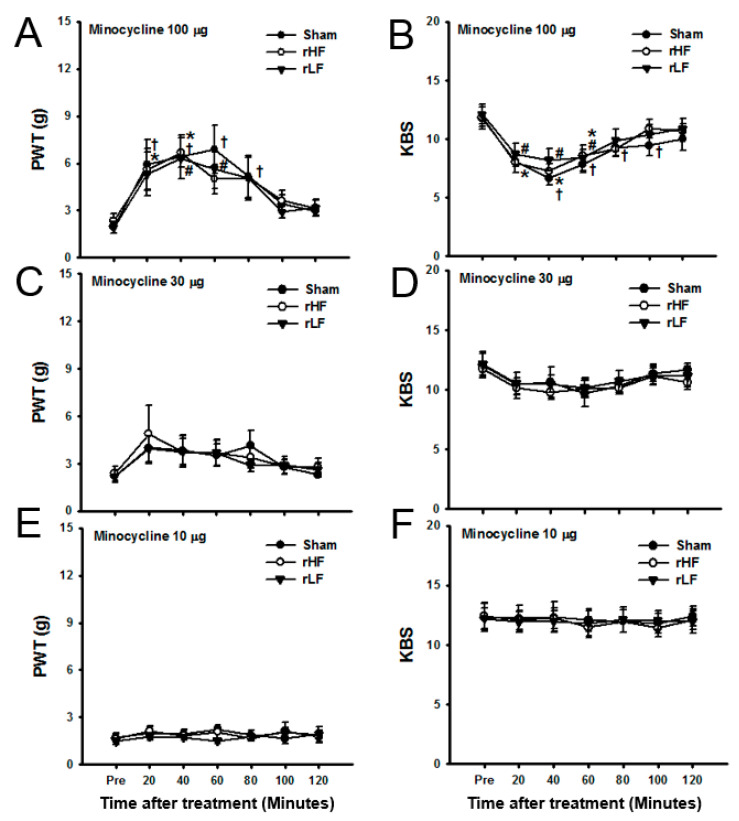
The effect of minocycline on osteoarthritic pain in transcutaneous electric nerve stimulation (TENS)-tolerant rats. The paw withdrawal threshold (PWT) (**A**) and knee-bend score (KBS) (**B**) of the ipsilateral side in TENS-tolerant rats significantly changed after intrathecal minocycline (100 μg) injection. The PWT (**C**) and KBS (**D**) of the ipsilateral side in TENS-tolerant rats did not significantly change after intrathecal minocycline (30 μg) injection. Moreover, minocycline (10 μg) did not change the PWT (**E**) and KBS (**F**) in TENS-tolerant rats. Asterisks (*) indicate values significantly different from the value before TENS in the repeated high-frequency (HF) TENS group. Number signs (#) indicate significant changes in the values compared with the value before TENS in the repeated low-frequency (LF) TENS group. Dagger signs (†) indicate significant changes in the values compared with the value before TENS in the sham TENS group.

In contrast with the effect of a single TENS application on glial expression in rats with OA [6], repeated applications of TENS failed to change the glial expression in the spinal cord. Moreover, this study investigated whether intrathecal minocycline, a microglia inhibitor, could reverse TENS-induced tolerance on OA pain. Rats exposed to daily TENS application for six days (TENS-tolerant rats) were used. To determine the effect of microglial inhibition on OA pain in TENS-tolerant rats, minocycline was intrathecally injected. In these rats, mechanical hypersensitivity in the hind paw and knee joint pain significantly changed after 100-μg minocycline treatment (Figure 4A,B) in each group compared to baseline values, but did not change significantly after the administration of 30-μg (Figure 4C,D) or 10-μg (Figure 4E,F) minocycline.

### 3.4. Effects of TENS with Microglia Inhibition on OA Pain in TENS-Tolerant Rats

**Figure 5:**
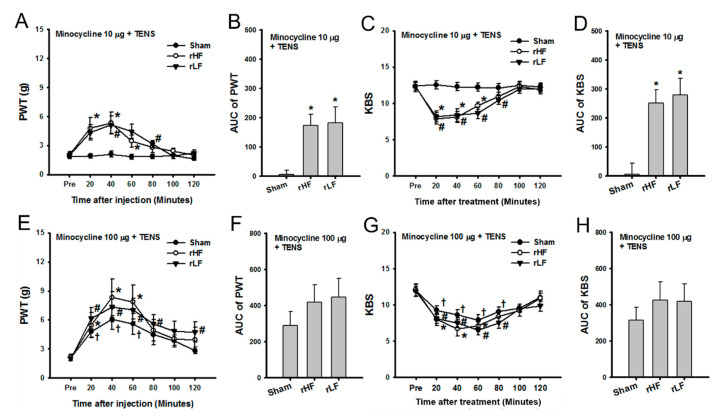
The effects of transcutaneous electric nerve stimulation (TENS) with minocycline treatment on osteoarthritic (OA) pain in TENS-tolerant rats (**A**) The paw withdrawal threshold (PWT) in TENS-tolerant rats significantly changed after TENS with minocycline (10 μg) treatment. The PWT significantly increased from 20 min to 60 min after high-frequency (HF) TENS with minocycline and 40–80 min after low-frequency (LF) TENS with minocycline compared with that before TENS with minocycline in TENS-tolerant rats. (**B**) After treatment, both the HF and LF TENS groups showed a significant increase in the overall effect on mechanical hypersensitivity of the hind paw compared with the sham TENS group. (**C**) The knee-bend scores (KBS) in TENS-tolerant rats significantly changed following TENS with minocycline treatment. The KBS significantly decreased from 20 min to 60 min after HF TENS with minocycline treatment and from 20 min to 80 min after LF TENS with minocycline compared with that before TENS with minocycline in TENS-tolerant rats. (**D**) After treatment, both the HF and LF TENS groups showed a significant increase in the overall effect on knee joint pain compared with the sham TENS group. (**E**) The PWT in TENS-tolerant rats significantly changed after TENS with minocycline (100 μg) treatment. The PWT significantly increased from 20 min to 60 min after HF TENS with minocycline and from 20 min to 80–120 min after LF TENS with minocycline compared with that before TENS with minocycline in TENS-tolerant rats. In addition, sham TENS with minocycline treatment significant increased the PWT from 20 min to 60 min. (**F**) After treatment, the HF and LF TENS groups did not show a significantly increase in the overall effect on mechanical hypersensitivity following OA compared with the sham TENS group. (**G**) The KBS in TENS-tolerant rats significantly changed following TENS with minocycline treatment. The KBS significantly decreased from 20 min to 60 min after HF TENS with minocycline treatment and from 20 min to 80 min after LF TENS with minocycline compared with that before TENS with minocycline in TENS-tolerant rats. Furthermore, sham TENS with minocycline treatment significantly decreased knee joint pain from 20 min to 80 min after treatment. (**H**) After treatment, the HF and LF TENS groups did not show a significant decrease in the overall effect on OA knee pain compared with the sham TENS group. (**A**,**C**,**E**,**G**) Asterisks (*) indicate values significantly different from the value before intervention in the HF TENS group. Number signs (#) indicate the significant changes in the values compared with the value before intervention in the LF TENS group. Dagger (†) signs indicate significant changes in the values compared with the value before intervention in the sham TENS group. (**B**,**D**) Asterisks (*) indicate values significantly different from the AUC in the sham TENS group.

This study investigated whether minocycline could reverse TENS-induced tolerance when co-treated with TENS. Minocycline (10 μg) was administered intrathecally, and sham, HF, or LF TENS was applied. The PWT in TENS-tolerant rats significantly changed after TENS with minocycline treatment. Mechanical hypersensitivity in the hind paw was significantly restored from 20 min to 60 min after HF TENS with minocycline and 40 and 80 min after LF TENS with minocycline compared with that before TENS application with minocycline in TENS-tolerant rats (Figure 5A). Sham TENS with minocycline did not change the PWT over time (Figure 5A). After treatment, both the HF and LF TENS groups showed a significant increase in the overall effect on hind paw pain compared with the sham TENS group (Figure 5B). The KBS in TENS-tolerant rats significantly changed following the application of TENS with minocycline treatment. Knee joint pain significantly decreased from 20 to 60 min after HF TENS with minocycline treatment and from 20 to 80 min after LF TENS with minocycline compared with that before TENS application in TENS-tolerant rats (Figure 5C). Sham TENS with minocycline did not change the KBS over time (Figure 5C). After treatment, both the HF and LF TENS groups showed a significant increase in the overall effect on knee joint pain compared with the sham TENS group (Figure 5D).

Minocycline (100 μg) was given intrathecally, and sham, HF, or LF TENS was applied in each group. The PWT significantly changed after the application of TENS with minocycline treatment. The PWT significantly increased from 20 min to 60 min after HF TENS with minocycline and from 20 to 80–120 min after LF TENS with minocycline compared with those before the application of TENS with minocycline in TENS-tolerant rats (Figure 5E). Moreover, sham TENS with minocycline treatment significantly increased the PWT from 20 to 60 min after treatment (Figure 5E). Regarding the overall effects of TENS with minocycline on mechanical hypersensitivity, the HF and LF TENS groups did not show a significant increase in AUC of the PWT compared with the sham TENS group (Figure 5F). The KBS in TENS-tolerant rats significantly changed following TENS with minocycline treatment. Knee joint pain significantly decreased from 20 min to 60 min after HF TENS with minocycline treatment and from 20 to 80 min after LF TENS with minocycline compared with that before the application of TENS with minocycline in TENS-tolerant rats (Figure 5G). Furthermore, sham TENS with minocycline treatment significantly decreased knee joint pain from 20 min to 80 min after treatment (Figure 5G). The HF and LF TENS group did not show a significant decrease in the overall effect on OA knee pain compared with the sham TENS group (Figure 5H).

### 3.5. Spinal Glial Changes Induced by TENS Treatment with 10-μg Minocycline in TENS-Tolerant Rats with OA

**Figure 6:**
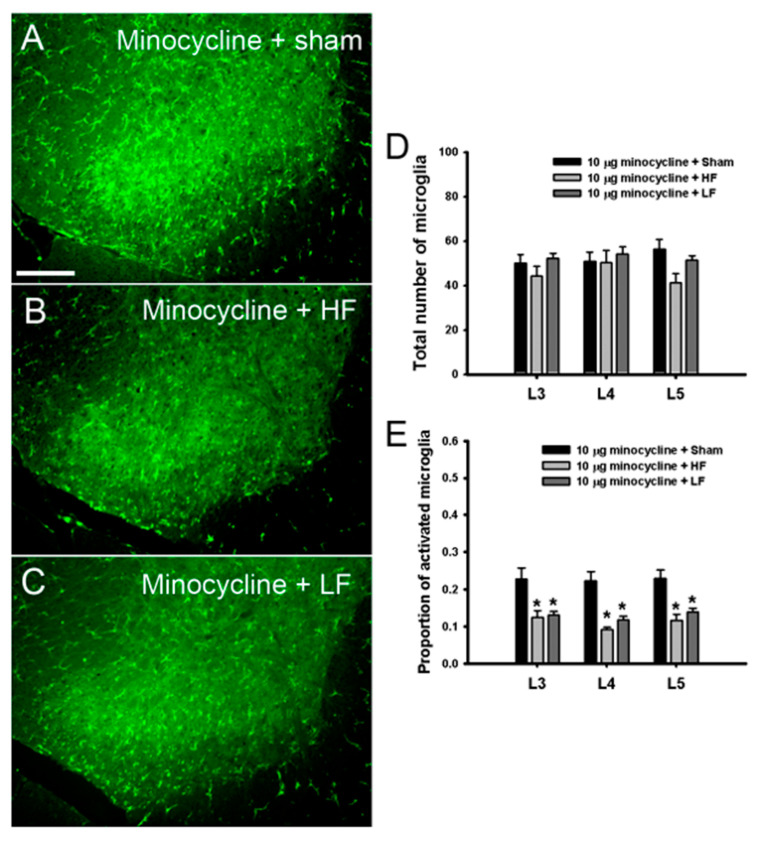
Changes in the expression of spinal microglia due to transcutaneous electric nerve stimulation (TENS) with minocycline (10 μg) in TENS-tolerant rats (**A**) The expression of activated microglia was exhibited after sham TENS with minocycline. High-frequency (HF) TENS with minocycline (**B**) and low-frequency (LF) TENS with minocycline (**C**) changed the expression of microglia from the activated state to the resting state. (**D**) HF and LF TENS with minocycline treatments did not significantly change the total number of microglia expressed in the spinal segments compared with sham TENS with minocycline. (**E**) Compared with sham TENS with minocycline, both HF and LF TENS with minocycline treatment significantly decreased the proportion of activated microglia in the L3, L4, and L5 spinal segments. Asterisks (*) indicate values significantly different from that in the sham TENS with minocycline group. Scale bar: (**A**–**C**) 200 μm.

To identify whether TENS with minocycline affects the expression of spinal microglia in TENS-tolerant rats with OA, the expression of microglia was examined in the TENS with minocycline (10 μg) group. HF TENS with minocycline (Figure 6B) and LF TENS with minocycline (Figure 6C) decreased the activated morphology of microglia compared with sham TENS with minocycline (Figure 6A). HF and LF TENS with minocycline treatment did not significantly change the expression of microglia in the spinal segments compared with sham TENS with minocycline (Figure 6D). Compared with sham TENS with minocycline, both HF and LF TENS with minocycline treatment significantly decreased the proportion of activated spinal microglia (Figure 6E).

## 4. Discussion

In this study, repeated applications of TENS decreased the analgesic effect on OA pain and failed to reduce the microglia activation in the spinal cord. Minocycline, a spinal microglia inhibitor, restored TENS-induced analgesia on OA pain and the TENS-induced inhibition of activated microglia in TENS-tolerant rats, suggesting that the development of analgesic tolerance due to repeated TENS applications affects the expression of spinal microglia in rats with chronic OA.

Chronic OA pain features an overlap between arthritic pain and neuropathic pain [36] and features of neuropathic pain in knee OA are associated with central sensitization [37]. Central sensitization induced by spinal glial activation can contribute to chronic OA pain [27,38]. A recent study has demonstrated that TENS significantly reduced OA pain and its effect was related to spinal microglial inhibition [6]. In this study, a single TENS application significantly decreased OA pain, but repetitive daily applications of HF (from day 5) or LF TENS (from day 6) decreased the analgesic effect of TENS on OA pain. The expression of activated microglia in the spinal cord following repeated TENS applications was similar to that in the sham group. The results of this study suggest that repeated TENS applications induced analgesic tolerance and failed to inhibit the expression of activated microglia in rats with OA.

Repeated opioid treatments decrease opioid analgesia in which glial activation affects development of tolerance [23]. In addition, the anti-opioid system contributing to opioid tolerance may be activated by the changed expression of factors released through glia [24]. Repeated opioid treatments may upregulate the N-methyl-D-aspartate receptor NR2A subunit by a brain-derived neurotrophic factor released from activated microglia [39,40,41] and the increase in glutamate levels in the synaptic cleft due to the decreased expression of glutamate transporter in astrocytes and neurons [42,43]. Considering that TENS-induced analgesia is associated with the release of endogenous opioids or the activation of opioid receptors in the central nervous system [7,8,9], the current results that the development of analgesic tolerance and no significant decrease in the proportion of activated microglia following repeated TENS applications may share the same mechanisms with those in opioid tolerance.

This study tested the effect of a microglia inhibitor, minocycline, in TENS-tolerant rats to investigate whether microglia inhibition restores the decreased effect of TENS by the development of analgesic tolerance. Intrathecal low-dose minocycline did not reverse the TENS-induced analgesic tolerance. Interestingly, when TENS combined with low-dose minocycline was applied in TENS-tolerant rats, the analgesic effect of TENS sealed by TENS-induced analgesic tolerance was restored. Moreover, the proportion of activated microglia significantly decreased, which means the restoration of microglial inhibitory effects of TENS.

A study has shown that glial activation contributes to opioid tolerance as a part of the anti-opioid system [24], and spinal microglial inhibition restores morphine analgesia in rats with morphine tolerance [18]. Considering that TENS-induced analgesia has mechanisms related to the activation of spinal opioid receptors [7,8], the analgesic effect of the combination of microglial inhibition and TENS may explain the attenuation of opioid tolerance due to the inhibition of activated microglia.

There are some limitations. First, a limitation of this study was confirming only the development of analgesic tolerance following repeated TENS application and its mechanism related to spinal microglia according to frequency. Second, small sample size of this study did not secure statistical power and may limit reaching conclusions in this study. To reach a general conclusion for these findings, with a suitable sample size, repeated electric stimulations using other stimulation conditions, such as increasing intensity or changing wave forms, which can prevent TENS-induced analgesic tolerance, should be tested, and underlying spinal microglial mechanisms according to behavioral results should be investigated.

## 5. Conclusions

This study showed that repetitive TENS applications could develop analgesic tolerance on OA pain, which is associated with the failure to inhibit activated microglia in the spinal cord. These results provide preclinical evidence for the clinical use of repetitive electric stimulation for OA pain control and for further studies.
